# Iatrogenic diaphragmatic hernia – a serious delayed complication: Three cases report and literature review

**DOI:** 10.1097/MD.0000000000045843

**Published:** 2025-11-07

**Authors:** Chengfeng Jin, Xiaohui Yang, Tiemei Han, Mengli Yu, Jiangwei Zhou, Yiyang Dai

**Affiliations:** a Department of Gastroenterology, The Fourth Affiliated Hospital of School of Medicine, and International School of Medicine, International Institutes of Medicine, Zhejiang University, Yiwu, China; b Department of General Surgery, The Fourth Affiliated Hospital of School of Medicine, and International School of Medicine, International Institutes of Medicine, Zhejiang University, Yiwu, China.

**Keywords:** endoscopic full-thickness resection (EFTR), hepatic radiofrequency ablation (RFA), iatrogenic diaphragmatic hernia (IDH), thoracic surgery

## Abstract

**Rationale:**

Iatrogenic diaphragmatic hernia (IDH) is rare, however, its incidence has increased with the growing number of thoracic and abdominal operations. IDH is a serious delayed complication that is easily missed and may lead to severe consequences. Recognizing and emphasizing the occurrence of IDH may help reduce its incidence. We report three cases of IDH from the past decade, each resulting from different operations, and managed with distinct treatments.

**Patient concerns:**

The first case occurred 4 months after a left upper lobectomy. Due to a lack of awareness, improper placement of a gastric tube resulted in gastric perforation.

The second patient presented with symptoms 10 years after endoscopic full-thickness resection of a gastric fundus gastrointestinal stromal tumor and was diagnosed 12 years after the operation. The cause was likely diaphragmatic injury during endoscopic full-thickness resection, owing to the proximity of the diaphragm to the gastric fundus.

The third case occurred 9 months after radiofrequency ablation (RFA) for liver cancer, which involved penetration through the diaphragm and caused iatrogenic damage.

**Diagnoses:**

The first patient was initially misdiagnosed at a local hospital and was finally diagnosed with IDH and gastric perforation using computerized tomography (CT), gastroscopy, and upper gastrointestinal radiography. The second patient was diagnosed by CT and upper gastrointestinal radiography. The third patient was diagnosed by enhanced CT.

**Interventions:**

Surgical repair was performed on the first and third patients. The second patient declined surgery due to advanced age and having adapted to the discomfort caused by IDH.

**Outcomes:**

The first patient recovered well postoperatively and showed no recurrence during the 10-year follow-up period. The second patient was experienced persistent discomfort, which did not worsen throughout the 5-year follow-up. The third patient also recovered well postoperatively and showed no recurrence during the 1-year follow-up period.

**Lessons:**

We emphasize that when a patient presents with relevant symptoms and significant unilateral diaphragmatic elevation is observed on imaging, the possibility of a diaphragmatic hernia should be highly suspected, and early diagnosis and treatment are essential. Furthermore, during operations that carry a risk of diaphragmatic injury-particularly hepatic operations – we recommend meticulous protection of the diaphragm to prevent IDH.

## 1. Introduction

Diaphragmatic hernia (DH) results from a combination of thoracoabdominal pressure gradient and a defect in the diaphragm. It can be classified as congenital, traumatic, pregnancy-induced, or iatrogenic.^[1]^ Iatrogenic diaphragmatic hernia (IDH) is rare but serious. Current literature on IDH consists primarily of case reports and small case series. Cusumano C et al^[2]^ summarized 28 studies involving 11,368 hepatic operations and reported an IDH incidence of 0.75% (86 cases). Misdiagnosis or delayed diagnosis of IDH can lead to serious complications, including organ necrosis and death.^[3]^ Any operation that injures the diaphragm may cause IDH. With the increasing number of thoracoabdominal operations, the incidence of IDH has gradually risen. Our hospital admits 1 to 2 cases of DH per year (excluding hiatal hernia), 3 of which were IDH over the past 10 years. Here, we report 3 cases of delayed IDH resulting from different operations. One case had rare and severe consequences, and another has not been previously reported in the literature.

## 2. Cases presentation

### 2.1. Case 1

A 55-year-old female was admitted to the emergency department with a 5-day history of nausea and vomiting, and a 2-day history of chest tightness, and shortness of breath. She had undergone thoracoscopic left upper lobectomy for lung cancer 4 months earlier, with a pathological stage of T1N0M0. She denied any history of trauma. Five days before admission, she developed nausea and vomiting, accompanied by left upper abdominal distension and pain. Gastroscopy at a local hospital revealed substantial food retention in the stomach, and an abdominal plain film revealed small air-fluid levels in the small intestine. This patient was initially suspected to have a small intestinal obstruction. She was managed with fasting, fluid resuscitation, and insertion of a gastric tube (placed 53 cm from the incisors). Two days before admission, she had developed chest tightness and shortness of breath. A subsequent chest CT scan revealed left-sided hydropneumothorax with significant compression of the left lung. A closed thoracic drainage was placed, and antibiotic therapy was intensified. However, her symptoms did not improve. And she was transferred to our hospital. On admission, her body temperature was 37.8°C, and other vital signs were stable. Physical examination revealed diminished breath sounds over the left hemithorax and tympany on percussion. The abdomen was soft and tender. The thoracic drain was patent, draining a small amount of gas and blood-tinged fluid. Laboratory tests revealed a white blood cell count of 12 × 10^9^/L, neutrophils accounting for 82%, a CRP level of 32 mg/L, and a serum potassium level of 3.45 mmol/L. At this point, the differential diagnosis included small bowel obstruction, and secondary pleural effusion due to infection. However, the atypical presentation and the history of diaphragmatic surgery raised our suspicion for an IDH.

**Figure 1.**
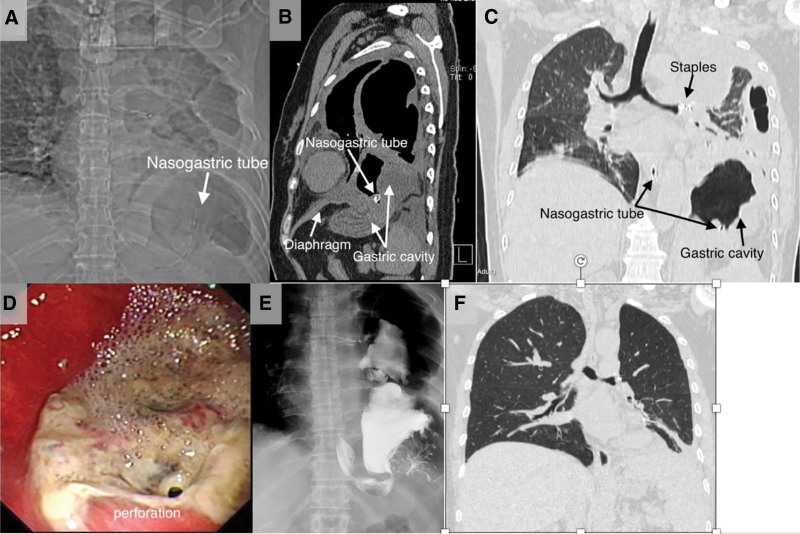
(A) The nasogastric tube was in a reverse “J” shape. (B/C) Gastric cavity passing through diaphragmatic defect. Left lung injury with pneumothorax. Partial stomach hernia into the chest cavity. (D) Gastric fundus perforation. (E) Upper gastrointestinal iohexol radiography revealed dissemination of the contrast medium in the thoracic cavity. (F) CT scan 3 months postoperatively revealed well recovery. CT = computerized tomography.

The chest radiography revealed the nasogastric tube forming a reverse “J” shape (Fig. 1A). CT revealed significant elevation of the left hemidiaphragm, left-sided pneumothorax, irregular compression of the left lung, and the herniation of the gastric cavity through a diaphragmatic defect (Fig. 1B, C). Gastroscopy revealed a round perforation in the gastric fundus (Fig. 1D). Upper gastrointestinal radiography with iohexol contrast revealed leakage of the contrast medium into the thoracic cavity (Fig. 1E). The patient was diagnosed with IDH accompanied by intrathoracic stomach, perforation of the gastric fundus, and left lung injury manifesting as pneumothorax and pleural effusion. The timeline of symptoms and diagnosis is shown in case 1, Table 1. She underwent transabdominal repair of the diaphragmatic defect, choledochoscopy-assisted intrathoracic repair, and a partial gastrectomy. A follow-up lung CT scan 3 months postoperatively revealed well recovery (Fig. 1F), and no recurrence was observed during the subsequent 10-year follow-up period.

**Table 1:** The timeline of symptoms and diagnoses for all 3 cases.

	Operation	Time of first symptom	Time of secondary symptoms	Repair surgery	Follow-up period (yr)
Case 1	Left upper lobectomy 4 mo ago	5 d before diagnosis	2 d before diagnosis	Laparoscopy	10
Case 2	Gastric fundus EFTR 12 yr ago	2 yr before diagnosis	–	None	5
Case 3	Right liver RFA 9 mo ago	1 d after diagnosis	–	Laparoscopy	1

EFTR = endoscopic full-thickness resection, RFA = radiofrequency ablation.

### 2.2. Case 2

An 80-year-old male patient was admitted to the hospital with a 2-year history of substernal obstruction sensation after eating. Twelve years ago, the patient had undergone endoscopic full-thickness resection (EFTR) for a gastrointestinal stromal tumor (GIST). Two years before admission, he began to experience symptoms including substernal obstruction, chest tightness, dull pain, heartburn, and belching. His medical history included hypertension and stroke. He denied any other history of trauma or other operations. Upon presentation, the initial differential diagnoses included esophageal motility disorders (such as achalasia), gastroesophageal tumors and even cardiac angina due to the patient’s advanced age and history of hypertension and stroke.

**Figure 2.**
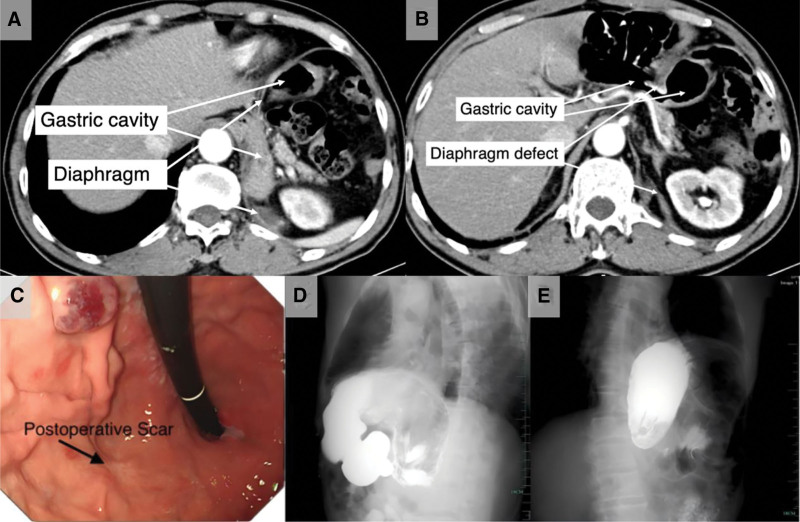
(A, B) Significant left hemidiaphragm elevation and defect. The herniated organs include partial stomach, small intestine, colon, spleen, kidney, and pancreas. (C) Gastroscopy revealed postoperative scar in the fundus, and this could be the location of the operation injury to the diaphragm. (D, E) Upper gastrointestinal radiography revealed a tortuous gastric cavity in the left thoracic cavity.

After admission, Electrocardiogram and troponin tests ruled out cardiac angina, and gastroscopy did not detect any disease such as gastroesophageal tumors, achalasia or reflux esophagitis. CT revealed significant elevation of the diaphragm with a large defect (Fig. 2A, B). Gastroscopy revealed marked distortion of the gastric cavity and postoperative scarring in the fundus (Fig. 2C). Upper gastrointestinal radiography revealed herniation of the gastric cavity into the left thoracic cavity, with the contrast medium passing into the small intestine (Fig. 2D, E). The imaging findings of a definitive diaphragmatic defect with intrathoracic migration of abdominal organs were pathognomonic for IDH. The diagnosis was IDH, likely attributable to diaphragmatic injury during the previous EFTR procedure, given the close anatomical proximity of the diaphragm to the gastric fundus. The timeline of symptoms and diagnosis is shown in case 2, Table 1. The necessity of surgery was explained to patients and his family. However, considering the patient’s advanced age, multiple comorbidities, 2-year history of stable symptoms, and overall clinical stability, the family declined surgery after thorough discussion. During the subsequent 5 years of follow-up, he patient continued to experience discomfort, but his symptoms did not worsen.

### 2.3. Case 3

**Figure 3.**
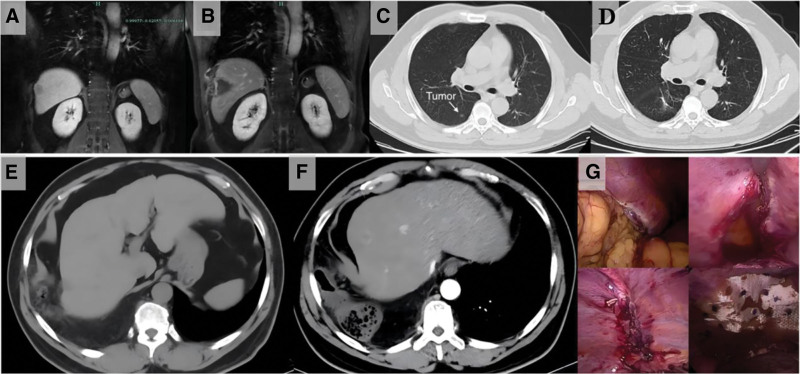
(A, B) MR images of hepatocellular carcinoma before and after RFA, revealed that the right diaphragm was injured. (C, D) CT images of the pulmonary adenocarcinoma before and after thoracoscopic wedge resection, revealed that it was not the location of the IDH. (E, F) Nine months after RFA, CT revealed a IDH with omentum and a small part of colon though the diaphragm defeat by RFA. The next day, the patient experienced right upper abdominal pain. Enhanced abdominal CT revealed a significant progress of the IDH. (G) Emergency laparoscopic surgery was performed to repair this IDH. CT = computerized tomography, IDH = iatrogenic diaphragmatic hernia, RFA = radiofrequency ablation.

A 55-year-old male with a history of hepatitis B presented an alpha-fetoprotein (AFP) level of 1713 ng/mL. Enhanced magnetic resonance imaging revealed a 2 cm nodule in segment 5 of the right liver (Fig. 3A). Ultrasound-guided biopsy confirmed hepatocellular carcinoma, and ultrasound-guided radiofrequency ablation (RFA) was performed (Fig. 3B). The patient subsequently underwent thoracoscopic wedge resection of the right lower lobe for a 6 mm nodule, which was diagnosed as pulmonary adenocarcinoma (Fig. 3C, D). The site of the pulmonary resection for cancer was excluded as the etiology of the IDH. Nine months later, follow-up chest CT revealed an IDH. One day after the CT examination, the patient experienced right upper abdominal pain. The differential diagnoses, including liver abscess, biloma, and acute cholecystitis, were considered. The emergency enhanced CT scan was crucial to differentiate these entities, as it clearly demonstrated the diaphragmatic defect with herniation and incarceration of the colon, rather than a complication solely within the liver parenchyma (Fig. 3E, F). The timeline of symptoms and diagnosis is shown in case 3, Table 1. It was fortunate that the IDH be detected before the symptoms appeared. Emergency laparoscopic repair of the IDH was performed; the diaphragmatic defect was repaired with interrupted nonabsorbable sutures and reinforced with a composite mesh (Fig. 3G). A follow-up abdominal CT scan 3 months postoperatively revealed well recovery. No recurrence was observed during the subsequent 1-year follow-up period.

## 3. Discussion

IDH is rare and often presents months to years after operation. Herniated abdominal organs may include the stomach, intestine, colon, spleen, kidney, omentum, and others. IDH can lead to serious complications including organs necrosis and death. Cusumano et al^[2]^ summarized 83 cases of IDH, 3 patients died before IDH repair (3.5%). The reported incidence of IDH varies, it was higher in those series involving diaphragmatic resection, which is a major risk factor for IDH, and the use of coagulation devices was another risk factor. Spellar et al^[4]^ reported that IDH is rare, but can be life-threatening due to bowel incarceration and strangulation, with an overall mortality rate as high as 31%. Kajal et al^[5]^ reported a congenital DH misdiagnosed as empyema with thoracentesis, leading to iatrogenic gastric perforation, a situation similar to our first case. Köneş et al^[6]^ analyzed 70 DHs, 9 cases (12.8%) were IDH, the postoperative mortality rate in their series was 7.1% (5 cases). In our first case, insufficient recognition of the disease and improper gastric tube placement resulted in gastric perforation, gastrothoracic fistula, complicating both the clinical presentation and surgical management. When a patient presents with both abdominal and respiratory symptoms along with radiographic evidence of diaphragmatic asymmetry, the possibility of DH should be considered. Early diagnosis and prompt intervention are crucial for improving patient outcomes.

Operations that cause diaphragmatic injury and/or increase the pressure gradient between the thoracic and abdominal cavities may lead to IDH. These include cardiac surgery,^[7]^ thoracoscopic diaphragmatic surgery,^[8]^ hepatic surgery, laparoscopic resection of GIST,^[9]^ pulmonary artery ligation,^[10]^ Nissen fundoplication,^[11]^ renal and pancreatic surgery,^[12]^ lateral anterior column realignment,^[13]^ splenic surgery,^[14]^ diaphragmatic tumor biopsy,^[15]^ pulmonary surgery, and even colonoscopy.^[16]^ The second case of IDH was diagnosed at 12 years after EFTR of a GIST, which, to our knowledge, had not been previously reported. The thin gastric fundus and the requirement for full-thickness resection during endoscopic operation might have resulted in inadvertent diaphragmatic injury. Over time, the persistent pressure gradient between the thoracic and abdominal cavities likely contributed to the development of IDH. The successful conservative management without surgery invites reevaluation the treatment strategies for IDH. We recommend that during EFTR, the electrosurgical knife should always be kept within the endoscopic field of view to avoid injury to extra-gastric tissues and organs.

The protective effect of the right subdiaphragmatic liver makes left-sided diaphragmatic injuries more likely to lead to DH. Congenital and traumatic DH occur more frequently on the left side, with a reported incidence of approximately 80%.^[1]^ In contrast, IDH occurs more commonly on the right side, largely due to the frequency of hepatic operations – including liver transplantation, partial hepatectomy, and RFA. Cusumano et al^[2]^ summarized 86 cases of IDH following hepatic operations, 79% (68 cases) of which occured on the right side. Esposito et al^[17]^ reported 3 cases of IDH, all on the right, with an incidence of 2.3% (3/131) following liver resection. Lee SW et al^[3]^ reviewed 45 cases and reported that the incidence of IDH after hepatectomy varied between 0.6% and 2.7%, depending on the extent and type of resection. among these, 38 cases (84%) were right-sided, 3 were left-sided, and 4 were unspecified. IDHs related to RFA for hepatocellular carcinoma have predominantly been reported as right-sided case reports.^[18–23]^ Tsunoda et al reported that laparoscopic RFA, or the use of artificial ascites might protect diaphragm from burn injuries during RFA.^[23]^ Diaphragmatic injury during hepatic operatios is the direct cause of IDH. Furthermore, the complications such as liver dysfunction (leading to malnutrition), cirrhosis (reducing the diaphragm’s protective support), and ascites (increasing abdominal pressure) are associated with a higher risk of developing IDH.

Once diagnosed with IDH, surgery is generally recommended.^[2,17]^ Most patients with IDH experience abdominal pain and respiratory symptoms. However, Esposito et al^[17]^ reported that 10.7% (3/28) of IDH cases were asymptomatic, while Cusumano et al^[2]^ summarized an asymptomatic rate of 6.9% (6/86). Our second patient lived normally without surgery. Possible explanations include: A large diaphragmatic defect may reduce the risk of organ incarceration due to the absence of a narrow hernia orifice; a new pressure equilibrium may become established between the pleural and abdominal cavities. We emphasiz that the treatment strategy for IDH should be carefully discussed with the patient and their family, particularly in asymptomatic cases, given that postoperative mortality can reach 7.1%.^[6]^ This case underscores a broader philosophical shift that is gaining traction across various surgical disciplines: prioritizing minimally invasive or noninvasive management and rigorously weighing the risks of intervention. Our findings in IDH, which caution against inadvertent surgical injury and highlight the viability of watchful waiting in selected cases, are strongly supported by evidence from other fields. A recent meta-analysis by Meng et al^[24]^ on degenerative meniscal tears found that exercise therapy provided equivalent long-term functional outcomes to arthroscopic partial meniscectomy, but was associated with a lower risk of detrimental long-term sequelae (osteoarthritis progression). This parallel underscores a critical surgical principle: the risks of intervention must be rigorously weighed against its benefits, and a conservative approach should be considered a viable first-line strategy whenever possible.

## 4. Limitations and strengths

Our cases are notable and distinctive in several respects. case 1 illustrates the complex and severe complications that may arise following misdiagnosis. Although surgery is typically necessary for IDH, the patient in case 2 – despite being diagnosed with a significant IDH – remained normal life over the subsequent 5 years without surgery. Case 3 demonstrates the potential for rapid progression of IDH, with symptomatic incarceration occurring within a single day. However, our ability to generalize these findings is constrained by the limited number of cases. We are unable to provide detailed epidemiological data on IDH incidence, as some mild cases may have gone undetected. Additionally, we lack systematic follow-up data on postoperative recurrence. Further studies with larger cohorts are needed to address these questions.

## 5. Summary

IDH is a rare but serious postoperative complication that requires prompt diagnosis and treatment to prevent secondary injury. When a patient presents with relevant symptoms and radiography reveals significant asymmetry between the left and right sides of the diaphragm, the possibility of IDH should be considered. Furthermore, we emphasize that during operations posing a risk of diaphragmatic injury – particularly hepatic operation – meticulous protection of the diaphragm is essential to help prevent IDH.

## Author contributions

**Conceptualization:** Chengfeng Jin, Tiemei Han.

**Data curation:** Chengfeng Jin, Xiaohui Yang, Tiemei Han.

**Formal analysis:** Chengfeng Jin, Mengli Yu.

**Funding acquisition:** Yiyang Dai.

**Investigation:** Jiangwei Zhou.

**Methodology:** Chengfeng Jin, Tiemei Han.

**Project administration:** Chengfeng Jin.

**Resources:** Chengfeng Jin, Xiaohui Yang, Jiangwei Zhou.

**Software:** Chengfeng Jin, Mengli Yu.

**Supervision:** Mengli Yu, Jiangwei Zhou, Yiyang Dai.

**Validation:** Jiangwei Zhou, Yiyang Dai.

**Visualization:** Mengli Yu, Jiangwei Zhou.

**Writing – original draft:** Chengfeng Jin, Tiemei Han.

**Writing – review & editing:** Chengfeng Jin, Mengli Yu, Yiyang Dai.
